# What Role for Law, Human Rights, and Bioethics in an Age of Big Data, Consortia Science, and Consortia Ethics? The Importance of Trustworthiness

**DOI:** 10.3390/laws4030515

**Published:** 2015-09-01

**Authors:** Edward S. Dove, Vural Özdemir

**Affiliations:** 1 J. Kenyon Mason Institute for Medicine, Life Sciences and the Law, School of Law, University of Edinburgh, Old College, South Bridge, Edinburgh EH8 9YL, UK; 2 Faculty of Communications and Department of Industrial Engineering, Office of the President, International Technology and Innovation Policy, Gaziantep University, Gaziantep 27310, Turkey; vural.ozdemir@alumni.utoronto.ca or vural.ozdemir@gantep.edu.tr; 3 Amrita School of Biotechnology, Amrita Vishwa Vidyapeetham (Amrita University), Amritapuri, Clappana P.O., Kollam, Kerala 690 525, India

**Keywords:** Big Data, Big Ethics, bioethics, commercial use, consortia ethics, consortia science, extreme centrism, global governance, health, trustworthiness

## Abstract

The global bioeconomy is generating new paradigm-shifting practices of knowledge co-production, such as collective innovation; large-scale, data-driven global consortia science (Big Science); and consortia ethics (Big Ethics). These bioeconomic and sociotechnical practices can be forces for progressive social change, but they can also raise predicaments at the interface of law, human rights, and bioethics. In this article, we examine one such double-edged practice: the growing, multivariate exploitation of Big Data in the health sector, particularly by the private sector. Commercial exploitation of health data for knowledge-based products is a key aspect of the bioeconomy and is also a topic of concern among publics around the world. It is exacerbated in the current age of globally interconnected consortia science and consortia ethics, which is characterized by accumulating epistemic proximity, diminished academic independence, “extreme centrism”, and conflicted/competing interests among innovation actors. Extreme centrism is of particular importance as a new ideology emerging from consortia science and consortia ethics; this relates to invariably taking a middle-of-the-road populist stance, even in the event of human rights breaches, so as to sustain the populist support needed for consortia building and collective innovation. What role do law, human rights, and bioethics—separate and together—have to play in addressing these predicaments and opportunities in early 21st century science and society? One answer we propose is an intertwined ethico-legal normative construct, namely *trustworthiness*. By considering trustworthiness as a central pillar at the intersection of law, human rights, and bioethics, we enable others to trust us, which in turns allows different actors (both nonprofit and for-profit) to operate more justly in consortia science and ethics, as well as to access and responsibly use health data for public benefit.

## 1. Introduction

Global health governance and reform, as well as debate on the linkages between science, bioethics, and human rights, have been in high gear the past several years [1-4]. Consider, as one example, the high-level plenary meeting of the General Assembly at the United Nations in September 2015 dedicated to approving the Sustainable Development Goals (SDGs) for the 2015–2030 term. The SDGs are envisioned as successors to the Millennium Development Goals (MDGs) that were agreed upon in 2000 and expired in 2015. The proposed SDGs have 17 overarching goals and an arguably ambitious set of 169 targets. The subject areas include urbanization, climate change, and the building of peaceful and inclusive societies that ameliorate human and environmental health. In part, the SDGs seek to reaffirm the “importance of freedom, peace and security, respect for all human rights…the rule of law, good governance, gender equality, women’s empowerment and the overall commitment to just and democratic societies for development” [5]. The SDGs are anticipated to be action-oriented but also aspirational, concise, and *global* in nature.

SDGs exemplify only one facet of the global interconnectedness of science and society in the new millennium, mediated through global governance structures and discourses. The “bioeconomy”, a term invoked by many countries and organizations such as the European Union and OECD to signify the exploitation of health data and other resources for large-scale life science and biotechnology projects and knowledge-based, commercializable and competitive products, has manufactured a source of hope (and hype) for economic stimulus and societal prosperity for rich and poor nations alike [6,7]. In the OECD’s report, *The Bioeconomy to 2030: Designing a Policy Agenda*, the bioeconomy is defined as “a world where biotechnology contributes to a significant share of economic output” and as “involv[ing] three elements: biotechnological knowledge, renewable biomass, and integration across applications” [8]. Indeed, the bioeconomy lies very much at the forefront of current global economic development, seen, for example, in the recent launch of the Precision Medicine Initiative in the US [9] and the 100,000 Genomes Project in the UK [10], not to mention similar developments in other regions [11-13].

The turn towards a bioeconomy is controversial [6,7,14-17] and raises critical questions. Is the bioeconomy, and the attendant strong appetite by innovation actors to “translate” basic research and raw data to knowledge-based products and applications, transforming law and human rights, as well as bioethics as a discipline, profession, and social movement? What capitalist or neoliberal discourses are conveyed, and what kinds of technological futures are forecasted [18]? Should significant amounts of public money be spent on molecular-focused studies rather than public health programs, or is “public health genomics” the best path forward for improving the population’s health [19,20]? What laws and regulations are (un)written, passed, and enforced (or court decisions rendered) to move the bioeconomy along, and what gets left behind (*i.e*., opportunity costs) in the process [21]? In what ways does the bioeconomy affect fundamental human rights [22]? How close have the ties between industry and academia become in the pursuit of “bench to bedside” translational science, have the ties caused damage to either or both sides, and is there collateral damage in society [23]?

Though the turn is recent, it has become evident that the bioeconomy brings new practices of knowledge co-production, and new understandings of about the nature of knowledge, to the fore. This is not to say that scientific practice and epistemology are only now experiencing structural change. Science, as in all disciplines, undergoes flux in paradigms and practices. Fields within science have grown “big” since at least the mid-20th century. In 1945, Vannevar Bush (former Director of the US’ Office of Scientific Research and Development, Dean of Engineering at MIT, President of the Carnegie Institute, and founder of Raytheon Corporation) famously wrote: “Science offers a largely unexplored hinterland for the pioneer who has the tools for his task. The rewards of such exploration both for the Nation and the individual are great. Scientific progress is one essential key to our security as a nation, to our better health, to more jobs, to a higher standard of living, and to our cultural progress” [24]. Bush encouraged the US federal government to spend millions of dollars on funding basic research; a few years later in 1950, the National Research Foundation was established to fund scientific projects across the country, in time to develop big machines and tools for the Cold War.

Further, as early as the 1960s [25,26], scholars invoked the term “Big Science” to signify society’s shift from individual or small group efforts to monumental scientific endeavors to seize its perceived societal aspirations, be it high-energy accelerators, manned space flight, rockets, or high-flux research reactors. These scholars contrasted “Big Science” to “little science” on a qualitative scale. It was not the size of the projects that determined “Big Science”; rather, it was the international, collaborative effort and networking of professional scientists and wealthy institutions who exchanged information formally and informally. Big Science was the evolution and maturation of certain scientific fields (indeed many fields could not and cannot become “big”). Little science, by contrast, was conducted often by single principle investigators (or scientific amateurs and enthusiasts) in smaller communities and with less infrastructure; these individuals engaged in small-scale, informal interactions and personal correspondence. Little science was characterized by heterogeneous methods and data and local control and analysis [27]. At the risk of presenting a somewhat Manichean dichotomy, these qualitative distinctions, we claim, still hold true today.

But, as we explain in this article, Big Science is undergoing transformation. The bioeconomic turn fosters hitherto unseen practices within Big Science, namely collective innovation and large(r)-scale *global* science that work alongside key actors—science-enabling ethicists—in a distributed, data-intensive and computation-intensive consortia system to solve large, intractable—and well-funded—scientific problems [27]. Such a transformation has consequences. As the former Editor-in-Chief of the leading journal *Science* opined in a 2012 editorial, small-scale artisan science can become directly threatened by the sheer power and growing scale of Big Science: “[…] the scale [of Big Science]…creates a constituency that makes these projects difficult to stop, even when there are clear signs of diminishing returns. […] Ensuring a successful future for the biological sciences will require restraint in the growth of large centers and -omics-like projects, so as to provide more financial support for the critical work of innovative small laboratories striving to understand the wonderful complexity of living systems” [28].

These new practices within Big Science, in turn, may be generating a paradigm shift in modes of reasoning about science and what science and data mean—in other words, ontological and epistemological changes. If the categories of “Big Science” and “little science” have remained with us since at least the post-World War II era, “consortia science” and “Big Ethics” (what we also call “consortia ethics”) are a relatively new emergence that spring forth out of the development—and institutionalization—of the bioethics discipline and profession, as well as out of laboratories and scholars working interdependently in publicly and privately well-funded consortia. Together, scientists and ethicists in these consortia work to actualize data-driven Big Science in an ostensibly ethical manner, but through particular methods.

**Figure 1:**
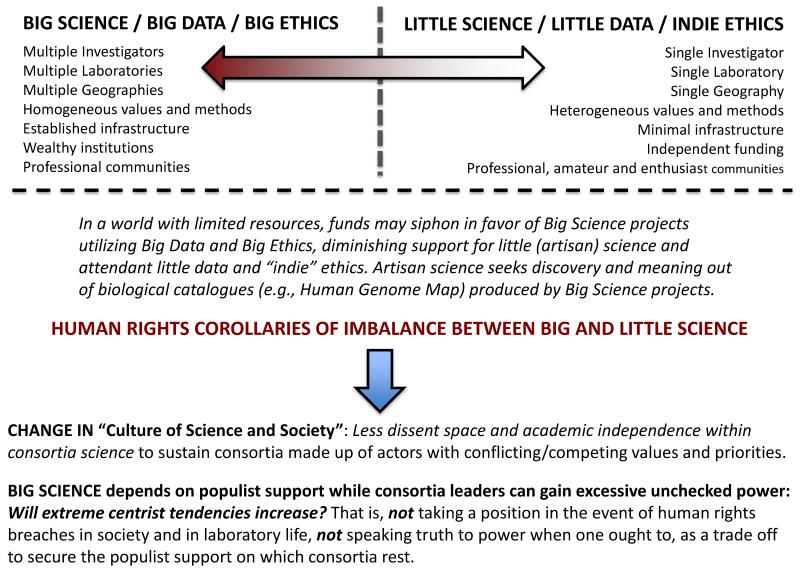
Mapping the linkages between consortia and artisan science, and bioethics and human rights in early 21st century societies. The categories of Big Science and little science are long-standing [25,26], a new development in the 21st century is the rise of consortia science and consortia ethics, characterized by well-funded research institutes, universities, medical centers, and public/private laboratories collaborating with each other and bioethicists across jurisdictions to share resources and capital with the ostensible aim of accelerating “ethical” scientific research discovery and translation for the bioeconomy. These forms of Big Science and ethics can lead to changes in the culture of science and society. Since Big Science “needs great public support it thrives on publicity” [25]; such populist support may increase extreme centrist tendencies.

These new forms and practices of science and bioethics also tend to depart from the previous models of small-scale or “artisan science” and ethics, which is signified foremost by greater independence, blue-skies thinking, and heterogeneity (Figure 1). If the traditional (and albeit mythic) role of both science and bioethics was to “speak truth to power” [29-33], the modern role of consortia science and ethics is to “propagate power as truth”—to build and maintain large consortia, and to keep innovation actors with disparate (competing, cooperating, or conflicted) values and methods somehow “glued” together for the sake of getting science and science-enabling bioethics done.

This renders subtle but fundamental value transformations within the human rights field and the bioethics profession. One such transformation is the ideology of “extreme centrism”, which we define as a fixed posture towards, or strong tendency to opt for, middle-of-the-road centrist normative positions that are the most popular (and usually self-serving), often divorced from contextual contingency and latent power. Extreme centrism is observed in the shadow efforts of key actors to sustain epistemic silence within the larger group, brooking little dissent and heterogonous diverse modes of reasoning, advocating and acting out a putatively neutral stance *no matter what*—even when principled, normative and possibly unpopular stances are called for—so as to keep a given consortium vibrant, well-funded, and innovative (or, in the modern mantra, “translational”).

As alluded to above, the 21st century also is marked by the increasing generation, collection, use and sharing of large-scale amounts of diverse data—what has been termed Big Data [34-37]. Governments, researchers, academics, industry, non-profit organizations, and citizens alike are forging links through the Internet and through real or virtual travel to share ideas and produce innovations. Interconnectedness and large-scale, Big Data-driven science bring many benefits, but also some predicaments, as highlighted above (Figure 1). Undoubtedly, sharing beneficial ideas and innovations can vastly improve human health and flourishing. Yet interconnectedness can also accelerate the risk of spreading disease—one reason why we now speak of “global health” and “global health diplomacy”. Big Data and interconnectedness can induce false positives and negatives in scientific discovery or new collaborators, who may or may not be trustworthy as the entry threshold to consortia science can be low (*i.e.*, by being at the right place at the right time). Big Data and interconnectedness can also lead to privacy intrusions, confusion of correlation with causation, as well as oppression [38-41].

Law, human rights, and bioethics play a strategic role in global science governance and in setting boundaries around the legal and moral behavior of actors in a globally connected ecosystem. But each of these three fields are normative and, in an era of “bigness”, be it in data, science, or ethics, they raise conceptual and practical challenges. Therefore, we should ask what kind of role each of these fields do and should play, particularly in instances where new modes of “doing” science and ethics emerge, a greater array of actors (with diverse motivations) come into the fold to make use of health data, and the unknowns of these modes can weigh as heavily as the knowns. Can law, human rights, and bioethics—separate and together—address the concerns associated with rise of consortia science and ethics, and how might they in turn be impacted by their rise?

In this article, we explore conceptually two manifestations of global interconnectedness in the health research context—the rise of consortia science and ethics, and the increasing commercial use (commercialization) of health data in the pursuit of a bioeconomy. We question what their impact might be on bioethics, law, and human rights, and how these fields in turn can address some of the tractable problems raised. We query how we may best address legitimate concerns about health data misuse, human rights, and conflicted interests, including asking whether law, human rights, and bioethics can serve as the primary and optimal tools for this new environment, so as to promote and protect a responsible, global health innovation ecosystem.

We offer a critical assessment of these three fields, and ultimately propose trustworthiness as an ethico-legal normative and universal *grundnorm* that can transcend and cut across all legal, human rights, and bioethical divides, serving as a central pillar at the intersection of these fields. Our purpose in raising these questions and posing alternative futures is not to negate the instrumental enabler role of consortia science and consortia ethics. They are here to stay, just as Big Science has been with us since at least the 1950s. Rather, we aim to ensure that little science, little data, and “indie” ethics can flourish, and that both they and their “big” and consortia-driven counterparts are grounded in virtue and justice.

## 2. The Emergence of Consortia Science and Consortia Ethics

Many actors around the world are now participating in what we call consortia science and consortia ethics [42]. On the science front, hitherto geographically and socially dispersed funding agencies, companies, research institutes, universities, medical centers, and public/private laboratories now collaborate with each other across jurisdictions on a formalized or institutionalized basis to share resources and capital with the ostensible aim of accelerating scientific research discovery and translating basic science into commercializable, competitive products for the bioeconomy. The names of these consortia-led initiatives are myriad, but examples include: the Human Genome Project, the Human Proteome Project, the Type 1 Diabetes Genetics Consortium, the International Serious Adverse Event Consortium, the Psychiatric Genomes Consortium, and the International Human Microbiome Consortium.

Consortia are modern developments in Big Science (and an underexplored issue in social science and bioethics). Big Science of the 20th century was characterized by formal or informal *communication* between professional scientists. Communication remains crucial, especially with the ubiquity of the Internet and social media, but Big Science of the 21st century is further characterized by formal or informal *agreements* between institutions, scientists, funders, governments, and other actors to act in unison in working on Big Science problems. The organizational structure has shifted from loose to solid, taking the form most often of a consortium, which means a group of people or institutions that explicitly *agree* to work together.

Similarly, hitherto dispersed bioethics scholars (or, before the field became a stand-alone discipline, philosophers, lawyers, and theologians) now collaborate not just to share ideas, but to build knowledge together as one well-funded integrated unit *with Big Science* [43]. Bioethics in much of the 20th century was noted for its analytically distant, critical stance towards science and medicine, holding “persistent skepticism about the moral authority of technical experts, whether that of the ‘bench researcher’, the clinical investigator, or the attending physician” [44]. Does this second-order, powerful monitoring role hold true today? We argue that it does, and should, but is diminishing in the face of the bioeconomy and consortia science.

The ELSI (Ethics, Law, and Social Implications of genetic and genomic research) [45], ELSA (Ethical, Legal, and Social Aspects of science and technology) and GE^3^LS (Genomics-related Ethical, Environmental, Economic, Legal, and Social) [46] research strands in academe reflect consolidated efforts with funders, government, industry, and natural scientists to define and pursue an agenda for ethics research in certain fields; the aim, ostensibly, is to align the interests of science and society [47]. This was first evidenced by the establishment of the ELSI program in the U.S. National Human Genome Research Institute in 1990. But whereas the original ELSI program was rather open to criticism of its source, the Human Genome Project, modern ELSI programs around the world now include everything from “integrated research”, *i.e.*, research grants with an ethics component “stapled on” to a scientific proposal to investigate ethical issues, to alliances that bring together established ELSI researchers from around the globe to co-produce certain enabling or instrumental ethical and regulatory tools with industry, funders, and the research community [47,48]. This emerging knowledge co-production links health actors (e.g., funders, natural scientists, ethicists, social scientists, and industrialists) in greater physical and epistemic proximity to integrate otherwise dispersed cultural practices, methods, epistemic cultures and norms, and data. The benefits of such closeness or “epistemic intimacy”—and there are benefits—have been noted and fit into the well-documented trend of large-scale systems science, Big Data, and public-private partnerships.

There is now a symbiotic relationship between science and ethics as the funding systems for consortia science also fund consortia ethics under the integrated research model. Big Science and Big Ethics become intertwined, and indeed, rarely can one now exist without the other. By contrast, in traditional small-scale “artisan” science, this is less of an issue, as artisan science and “indie” bioethics research almost always pursue different funders and agendas, lending ample critical space for bioethicists to critique, and for scientists to engage in blue-skies exploration (Figure 1). In consortia science and ethics, the blending of agendas can enclose the field of vision for all actors involved, and we ought therefore to question whether bioethics may lose its cutting edge or the critical epistemic distance necessary to analytically examine emergent issues in science.

Certainly, bioethicists will continue to have varying attitudes and ideas, yet consortia science encourages convergence, not divergence, of ideas, and bioethicists attached to Big Science projects often focus on issues set by science and industry [49]. Consortia ethics encourages orientation towards the consequences (impacts) of the research and development process, which can mask underlying political interests and disproportionately serve the interests of science [50]. It can lead to “compressed foresight” [51], where specific modes of reasoning are used “to produce seemingly accurate representations of a future, which leave to one side uncertainty and contingency” [47]. There are second-order consequences to consortia science, consortia ethics, and epistemic intimacy. What may be overlooked in the discourse for “integration” between consortia science and ethics is that the crucial analytical distance and independence of humanists, social scientists, and ethicists from natural scientists can be diminished or lost. In effect, this raises the potential for blunting the cutting edge analytical independent scholarship that actors have hitherto maintained to unpack scientific practices, thus lending, as documented as early as the Human Genome Project, a more passive “science enabling” (*i.e*., how can we help?) instrumental role for bioethicists [52-54].

Literature in organizational theory suggests that with increasing size of scientific teams, there is a bureaucratic hierarchy emerging that can lend excessive unchecked power to consortia leaders compared to smaller-scale science and single-laboratory investigators [55]. Hence, what we are witnessing with the rise of consortia science is, in part, an increase in the magnitude of some of the older problems in the organization of science and its bioethical aspects. However, in a world with limited financial resources, this massive restructuring of science through consortia and large-scale projects is also changing the culture of bioethics in that the “enabler” ethos is prevailing over and above other normative choices and agendas, in particular, (a) the opportunity costs of Big Science over small artisan science; and (b) the increased risk for complacency in the face of human rights breaches in laboratory life and in society, and diminished tendency for speaking truth to power to remedy or alleviate human rights breaches.

To be clear, and emphasize again, our point is not to denigrate either consortia or artisan modalities. In our view, both have their benefits and drawbacks, and generally speaking, we advocate greater international collaboration and data sharing as a principle. Indeed, it is possible to have an alliance or research consortia that is legally and ethically robust (and trustworthy, as we describe below) because it allows for open deliberation and participation, and encourages free, diverse thinking. Instead, our point is that consortia science and consortia ethics too often are treated as ends in themselves, or as inherently virtuous entities. While international collaboration and data sharing are valuable and can accelerate knowledge production and improve well-being, we must consider what else it can do.

We should be equally mindful of the potential drawbacks of small-scale artisan scientists and ethicists. Though generally they are more heterogeneous in their values, they might nevertheless, through ostracism and a desire to forge capital, become more ideologically united (be it through Marxism, feminism, environmentalism, communalism, *etc.*) and thereby closed to alternative ideologies. They may also become so removed from open communicative channels that whatever data, innovations, or knowledge are produced are disjointed or taken up by no one, which raises the question of what is the point if no one is listening. Surely the goal of small-scale science and ethics is not to become an echo chamber and serve no one but the self-gratifying ends of laboratory life and ethics analysis isolated from society. So, in either scenario of big or little science and ethics, there are benefits and predicaments. As we stress in the sections below, if transparency equates to both open dialogue (not unidirectional information dissemination) and deep participation, then it obligates artisan actors to reach out to broader audiences, and it obliges consortia actors to allow an indie spirit to flourish.

This said, our focus here looms larger for consortia science and ethics. New meta-issues for bioethics (an “on-frame” bioethics) should be developed by “indie” bioethicists and social scientists to consider how bioethics as a discipline, profession, and social movement may be changing in this era of big data-intensive consortia, and what impact consortia might have on law and human rights. Foremost, we query whether consortia could change the culture and social fabric of science and society in subtle, possibly harmful ways.

As one example to ponder, the saturated collaboration and integration/translation discourses attached to consortia can smother any “rocking of the boat” and encourage an ideology of extreme centrism [42]. The concept of “extremism” is historically and fashionably linked to stances situated at the polar ends of a distribution for a given social norm. However, as consortia science and attendant bioethics practices play a largely “science enabler” role together, this presents a veritable risk for remaining silent in the face of threats to human rights, so as to not rock the boat, or maintain the populist support on which various consortia rest.

In a climate of extreme centrism, voices and ideas on the tail ends can be squeezed out through controlled communicative processes, dismissed as radical and unsavory, and castigated as destabilizing threats to the bioeconomy. Consortia science and ethics can push for an ephemeral, almost sub-conscious ideology of bland quietude and platitudes so as to keep the consortia of diverse peoples together, at least for a period of time, *at all costs*. One potential consequence of extreme centrism, however, is an acute lack of open normativity and blue-skies thinking, precisely when openness is needed. Extreme centrism can even mutate into concretized non-normativity (*i.e*., avoiding normative stances), particularly if a consortium is led by the same group over a long protracted period due to weak governance structures—a kind of epistemic “locked-in syndrome”.

The real concern, then, is that extreme centrism and the risk of entrenched non-normativity can lead to ethical and legal blind spots, including violations of human rights, damaging the ontological and epistemological functions of science and ethics. Can the no-holds-barred maintenance of these consortia science and ethics structures take precedence over certain values we take to be universal and inviolable to humankind? If consortia science and ethics smother normative exploration and deliberation for the immediate aim of keeping diversity aligned, what might this do to justice and to virtues such as trustworthiness in the long-run?

## 3. Whither the Integrity and Independence of Scholars?

Consortia science and ethics and the ideology of extreme centrism can generate conflicted interests and challenge established human rights, including privacy and freedom of thought and expression, *i.e.*, the pursuit of truths through the marketplace of ideas. Consider, for example, the uniform requirement by credible peer-reviewed journals and the International Committee of Medical Journal Editors from authors of submitted manuscripts to assert that “the sponsors did not influence the design, collection, analysis and interpretation of data, the writing of the report, nor the decision to submit the paper for publication” [56]. It is generally expected that a firewall erected between the authors and their sponsors (or consultants paid by the sponsors) will ensure academic independence and prevent any undue influence over researchers to advance the sponsors’ interests. Absent such a firewall, fires can erupt: the ethics and health sciences literature is replete with evidence showing that privacy and the freedom to think and express can be severely undermined [57-61].

In the current age of consortia science and consortia ethics, researchers are often required to meet with their sponsors at predetermined intervals (*e.g.*, every quarter) for milestones and progress reports. Under the guidance of sponsor-paid consultants, these sponsors measure and decide on researchers’ progress. This practice resonates across academe. Consortia science, like much of higher education, has become awash in administration and bureaucracy. As Walsh and Lee have noted: “Rather than a focus on an individual’s lab bench, scientific work increasingly takes place in a setting that more closely resembles a small ‘factory’ or ‘quasi-firm’, run by a ‘small businessperson’ lab director” [55]. In network-driven Big Science, scientists and their bioethicist counterparts embedded in research teams are routinely and continuously monitored by a Scientific Advisory Board of the Big Science funders (e.g., [62]). These teams must submit to quarterly reporting of project milestones, deliverables, and budgetary expenses, ostensibly to achieve the (often translational and market-driven) research product goals beyond regular academic publications and knowledge production.

The obligatory condition to be measured and marked, coupled with the threat of withdrawn funding if the “performance metrics” are weak, signals a sharp departure from the past schemes of research grants where researchers were traditionally assured that their grant funding would continue within the specific term of their award. Many consortia now depend on the process-driven, metric-filled progress report model, a de facto “live contract model” whereby researchers are incessantly nudged to manufacture “products”, spawning a hypercompetitive numbers game for the most quantity of publications or tools, with less attention paid to their quality [63,64].

With this tight grip on the direction of science *and* ethics, scientists and bioethicists may, on the one hand, potentially reduce research waste and boost research performance. On the other hand, this may reduce the motivation to seek long-term project goals and diminish academic independence and the typical blue-skies research pursued by small science projects (let alone painstaking works at improving the quality of science that demand long-term investments). Because bioethicists can be connected to Big Science teams, such close monitoring and market orientation of research products from consortia science can also compromise the independence of bioethicists and their willingness to state opinions or findings that do not invariably bode well with the populist norms of the times. Attention of the innovation actors becomes shifted towards immediacy and short-term, quick gains. Foregone are more fundamental bioethical critiques that examine the epistemology of bioethics inquiry beyond an enabler/facilitator role given to bioethics by Big Science.

In such a climate, researchers’ and sponsors’ frenetic anxieties to generate outputs, be they publications or commercializable “products”, can detrimentally impact intangible values and interests such as privacy, publication ethics standards, and the public trustworthiness of the health research enterprise at large. Looking further, because researchers are also being asked to work harder to market their research data towards commercialized products, the privacy protections assured by normative guidelines such as the Declaration of Helsinki [65] to research participants and patients can be at greater risk, along with the researcher’s right to think and express freely. Yet, herein we can query whether human rights as currently structured go far enough both in recognizing the differences in individuals, who are suspended in a dense, dynamic web of relations, and in establishing the corresponding *duties* of actors to ensure that conflicts of interest are avoided and freedom of thought and expression is maintained [66,67]. It is an open question regarding how well can we rely on human rights to monitor actors (including consortia ethicists), hold them accountable, and ensure they actively engage in facilitating a responsible innovation environment [42].

In sum, the rise of consortia science and ethics is neither good nor bad *in itself*, but we should be cautious about how this movement is structured and governed, and the power that can be wielded over alternative modalities of science and ethics. We should question whether precious societal values might be lost in the march towards consortia science and ethics, including trustworthiness, and whether human rights (to say nothing of law and bioethics) can adequately assuage these concerns. We address these questions in the specific and timely context of commercial use of health data.

## 4. Health Data for All Seasons and Motives: Commercialization and Social Construction

Health, as both topic of concern and phenomenon represented in the environment and in individuals, has benefitted from global interconnectedness. The various and growing uses of health *data* have emerged as a focus of attention by many stakeholders—both public and private—who hold numerous motivations, some more transparent than others, for seeking to exploit them. Data are the currency of the 21st century, and health data are particularly valuable.

Some uses of health data, particularly when they are tied to an individual or community through identifying characteristics, can raise ethical conundrums and political controversies. Health data are treated both in law and ethics as “sensitive”, worthy of higher protection than other data types because of their inherent connection to the most deeply personal aspects of our lives. Naturally, people want to know that if data connected to their health status are collected by an external actor, that data will be safeguarded and not used in ways contrary to their interests and expectations or their explicit permission. We ask the same sorts of questions more abstractly: who collects health data, who determines what can be done with them, and what limits should be set on access and use? If these questions and ensuing answers are black-boxed—if people are excluded or sidelined from adequately deliberating on the motivations and merits of exploiting health data—scandal can erupt, and basic bioethical and human rights tenets can be violated. Let us consider one especially contested use—that of the *commercial use* of health data—which broadly defined can mean the access to or use of health data by for-profit companies for one or more profit-oriented purposes, including for insurance, employment, research, marketing, and health and social care.

The topic is of high concern to many publics. This is particularly the case when health data are personally identifiable and thus their use can lead to direct impact on the individuals or community connected to them, but this does not necessarily dissipate even if the data are anonymized or coded [68]. For evidence of this concern, we need look no further than coverage in newspapers, public opinion polls, or some of the highly accessed blogs [69-71]. Academic research, too, has noted concerns about potential commercial use of health data [72,73]. To take the example of a recent public scandal, the “care.data” program run by NHS England to extract, aggregate, and release data from primary care records and widen the range of data collected from secondary (hospital) care, became a black eye for the UK government in 2014 due to public concerns about the varying uses of NHS England patient data by undisclosed or under-disclosed actors, including the commercial sector. Though care.data was legal, it was not ethical, and no genuine attempt was made to elide the two fields through warrants of trust [74]. As a Nuffield Council on Bioethics report aptly notes, the scandal “highlighted the absence of governance arrangements to negotiate this difference [between ethical and legal norms], and raised questions about how the rights of individuals were respected. Failure to attend to these prospectively led to ad hoc policy changes and a damaging loss of public and professional trust” [75].

**Figure 2:**
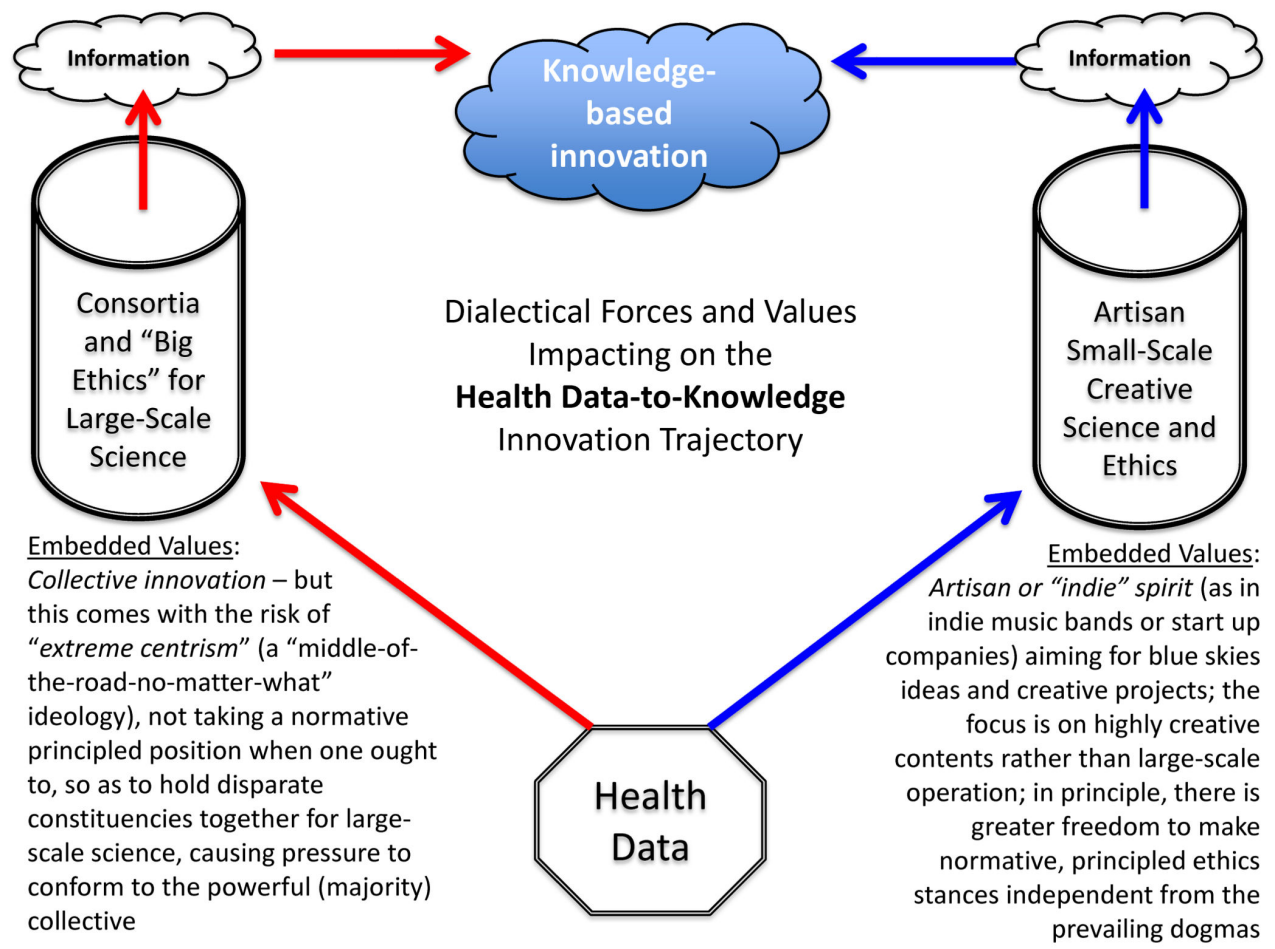
Dialectical forces and values impacting on the health data-to-knowledge innovation trajectory. Data, especially health data, are a key component fueling the bioeconomy. Increasingly, for-profit entities exploit these data and operate side-by-side other actors in large-scale science consortia. Small-scale, “artisan” science teams also exploit health data. Both churn these data into information and knowledge-based innovations. Ethicists play a role in this trajectory, either by examining the scientific practices of these actors and commenting on the bioethical implications, or by working alongside scientists to enable them to realize their innovations in an ostensibly ethical manner. However, the embedded values of these consortia and artisan small-science endeavors can differ. We claim that consortia may knowingly or unknowingly adopt an ideology of “extreme centrism”, while artisan small-science endeavors strive to maintain an “indie” spirit.

Commercial use of health data is an issue that incorporates global-local (“glocal”) tensions (consider for example, use of patient data in rural India by a multinational pharmaceutical company), law, human rights, and bioethics (Figure 2). Given its multi-disciplinary and sociopolitical ambit, robust engagement of publics and deliberation in the open should be encouraged. As we operate in an interconnected world increasingly dominated by powerful actors such as those within consortia, second-order narrators (on-frame observers) [76,77] should create agoras of truly open discussion about *what values are at stake*, and what *approaches* might best elide responsible uses of health data with respect for the morally relevant values of those whose data may be exploited.

Broadly speaking, we concur with those who are cautious, if not concerned, about private companies using health data collected from individuals either within or outside of healthcare systems. There is some evidence that points to a link between public confidence in the governance of health data protection and the mechanisms by which companies can access and use health data [78]. But just what are we to make of this finding?

First, public confidence cannot be manipulated like a lever, especially in the absence of clarity on the terms of the debate. There is a surprising and ongoing lack of precision around key terms, namely “commercial use” and “health data”, a lack of precision that is partly our own failing in the academic community *to ask the right questions before seeking (and such that we can seek) the right answers* [79]. We cannot begin to identify concerns (and validate those concerns) and offer solutions (and validate those solutions) about commercial use of health data until we expound on what we mean by “commercial”, “access/use”, and “data”, among other primary questions. If the law craves clarity and consistency, we must first establish clarity in what we are analyzing.

Consider the term “commercial”. The authors in one article addressing the topic emphasize the difficulties in clearly distinguishing between “commercial” and “non-commercial” research in the health sector [78]. Indeed. To bunch kaleidoscopic profit-seeking entities under one “commercial” umbrella, particularly in an era of public-private partnerships and consortia science (*i.e*., a melding of motivations and profit), is to merely obfuscate the debate and surely does not accurately reflect the veritable underlying, nuanced concerns of publics. Not all commercial entities are alike, especially in the health context. A family-run, privately held employer is distinct from a publicly traded life insurance provider, just as a multinational drug manufacturer is distinct from a search engine provider or a Big Data analytics-driven marketing company. Nor are any two companies within these varying commercial types the same. Many publics, we would venture to surmise, are *not* against profit-making entities using health data per se, though they may be concerned about some excessive profit or the exploitation of health to serve a purely profit-seeking motive. Rather, we would surmise that *some* commercial entities are viewed with more skepticism with others, just as we view certain government entities (e.g., a national security agency) with more skepticism than others (e.g., a public health agency). The strength of an entity’s reputation, based on social context and a long record of demonstrable virtue or vice, no matter the general sector, can significantly impact public acceptability and trust.

Additionally, there are various meanings of “access” and “use”, and the word “data” itself is subject to varying interpretation. Data—which comes from the Latin word *dare*, meaning “to give” (by nature), an irony because data are actually “taken” by humans and technologies—are socially constructed phenomena. They are inherently incomplete and selective precisely because they are merely abstractions of phenomena—chosen, measured, recorded, and interpreted in some way by some actor [80]. Data possess materiality, and have themselves become a liquid commodity in an age defined by the power of information (the “Information Age”), and they are a symbol of varying cultural meaning. Health data are universally valuable, but certain types may be more valuable in some places than others. The cultural meaning ascribed to health data will turn to some degree on whether the data are identifiable, meaning whether they can be traced to an individual or community, or whether they are anonymized such that they hold narrower meaning and value. But, it is a question of degree, not absolutes. No technological process performed on health data to remove any potential for re-identifiability is sufficient to wipe clean their cultural meaning, and thus cultural value—to say nothing of their monetary value.

This call for terminological precision leads us to a further claim: it is quite possible for injustices to be latently embedded in the choice of terms used to frame the discussion. How we ask the question determines the answer. The broader the terms, the greater risk of latent and black-boxed political subtlety. “Concerns about commercial use of health data” is an example, and our broad-brush use of this term has been deliberate to illustrate the point. It is abstractly framed, and abstract concerns labelled against abstract entities cannot lead to transparent and practical solutions offered by accountable actors in any governance system. If we acknowledge that publics are right to question the motivations behind use of health data by for-profit companies, we must formulate primary, specific, *ex ante* questions that unpack the concerns, working upstream to question the motivations and practices of actors and the just/unjust workings of data that have been “taken” by for-profit actors for multiple purposes. Each company, each dataset, and each purpose and process for access and use pose different issues.

Addressing commercial access to and use of health data, *holus bolus*, is nothing short a Sisyphean task and its framing a confidence trick. Until we first put each of these terms under a “political science microscope” to accurately source the actors, narrators, and problems, and to determine whether law, human rights, or bioethics can provide sufficient help, we cannot proceed to develop credible answers. The right answers are contingent on the questions we ask and the problems we uncover. We have asked questions that we hope delve deep into the heart of matter—a matter of increasing importance in consortia science and ethics. We now turn towards our claim that trustworthiness should serve as a central pillar at the intersection of law, human rights, and bioethics, as it allows different actors to access and responsibly use health data for public benefit.

## 5. Taxonomies, Trustworthiness, and Transparency

The commercial use of health data can be divided into a tripartite taxonomy of concerns: (1) *data misuse*, such as privacy intrusion, increased cost through price stratification, and economic or employment discrimination; (2) *profiteering, i.e.*, exclusive private benefit without any public benefit; and (3) *commercialization, i.e.*, corruption of the public service ethos of an organization such as the NHS [81]. This helps clarify what could otherwise remain vague qualms expressed by a phantom public. It also helps point to paths of remedy. But let us reflect on the driving question behind these qualms: If the common aim of a health governance system is to safeguard public confidence in appropriate uses of health data, how might we effectively address these concerns? This brings us back to our discussion of consortia science and ethics.

If the primary concern by publics is a perceived or real fear of misuse of health data for untoward ends, such fear, we think, rests on the use of data in a *non-transparent and unaccountable manner*. This fear, however, likely transcends the commercial space and can permeate all sectors, including government and the non-profit sector (to again cite the example of a national security agency, but also a multinational non-profit agency whose budget may exceed a country’s entire GDP). All the more reason then, why imposing sector-specific controls, in this case on the profit-seeking sector, will unlikely deliver good data governance, especially if those controls are imposed solely through hard law or regulation. The limits of law in health research regulation are well documented (e.g., [82-84]). As we explain further below, any approach that regulates commercial use of health data per se and (solely) through the force of law will be sub-optimal because law cannot in itself promote the conditions necessary and sufficient for public confidence, nor can it address satisfactorily all the *morally* and *socially* relevant interests of the diverse actors.

**Figure 3:**
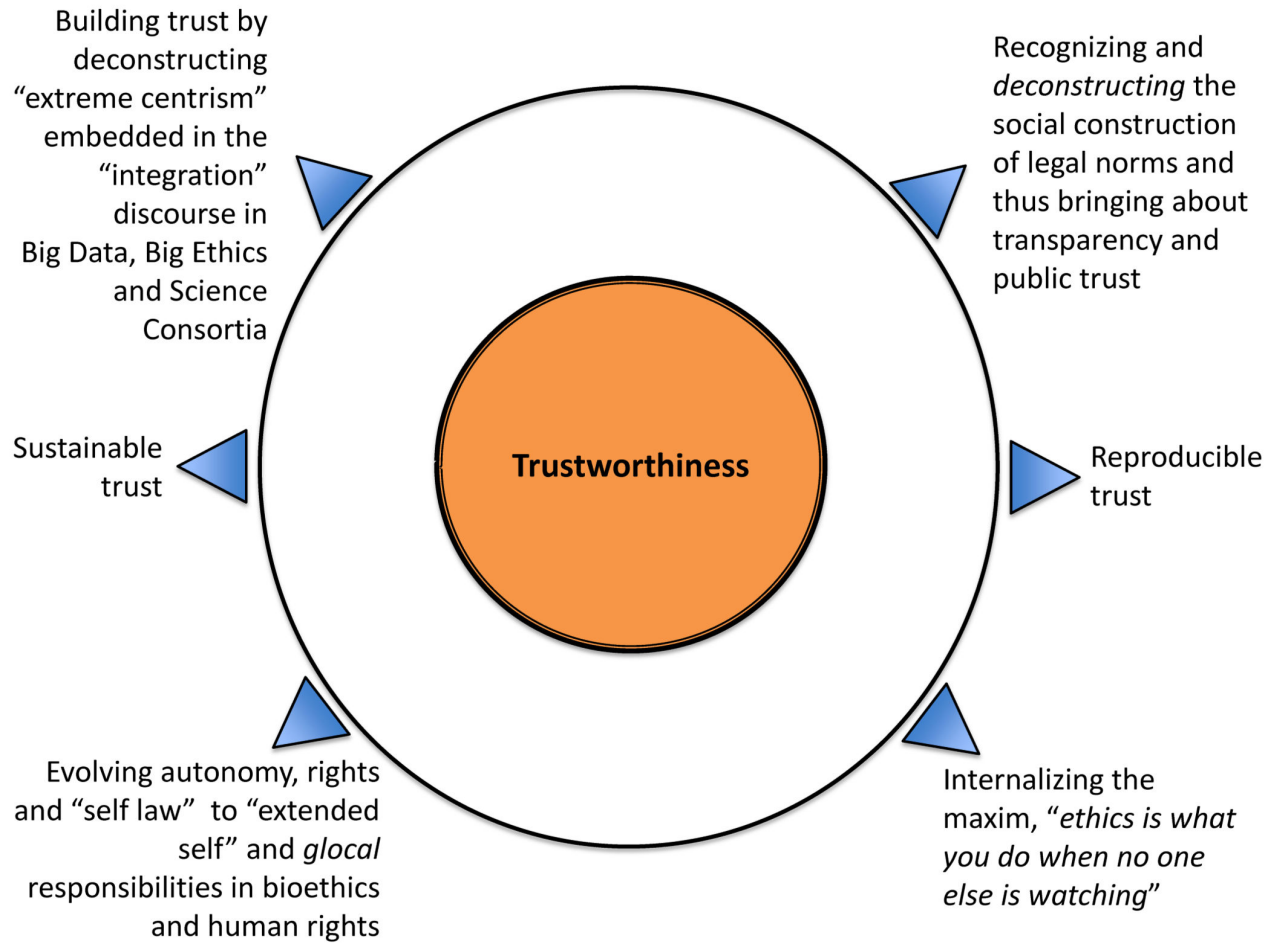
Elements of trustworthiness as a *grundnorm* for promoting public trust in consortia and health data exploitation by various actors. Ethically and legally just consortia, and responsive and responsible information governance, must be expressed and sustained through trustworthiness, which we define as the identification and realization of the conditions foundational to establishing and maintaining repeated and reproducible trust in another person, in a system, or in a practice. Trustworthiness is composed of several attributes, including transparency and accountability.

Instead of looking to law as a panacea for the concerns we have identified with consortia science, consortia ethics, and commercial use of health data, we should look to norms that that guide virtuous behavior. Foremost, ethically and legally robust consortia and responsive and responsible information governance—that is, systems that protect and promote the responsible and ethical stewardship of research and data—must be expressed and sustained through *trustworthiness*, which we define as the identification and realization of the conditions foundational to establishing and maintaining *repeated* and *reproducible* trust in another person, a system, or a practice. Trustworthiness is the basic ethico-legal norm—the *grundnorm*—we need in health research regulation and consortia science and ethics to promote public trust and confidence in systems that can and should allow responsible commercial use of health data (Figure 3).

How might we work towards good information governance that *mitigates* (but does not “avoid”—as that is an impossibility) the potential for misuse of health data in an era increasingly marked by consortia science and ethics, regardless of whether the misuse is by companies, government agencies, or other entities? Some might say a suitable path is instituting an option of individual opt-out for uses of one’s data, or for data controllers and processors to provide more information about what is done with health data (in other words, the purposes and actors behind access and use), or forming an effective independent “watchdog” or “independent review group” capable of protecting individual and public interests.

These are all more-or-less sensible options, but we question whether they constitute necessary and sufficient elements to establish and maintain trustworthiness. Thinking globally, watchdog committees, in particular, may not represent a locus of ethical responsibility and respect for a community. Instead, we propose one foundational element of trustworthiness, an element that must be designed correctly: *transparency of processes, particularly about all envisioned health data uses*. Let us be clear: transparency *cannot* be instantiated simply through unidirectional information dissemination. This is not transparency. Transparency is about creating the capacity for the availability, accessibility, and comprehensibility of information, which may be communicated across a network, from node to node. A transparent system is one that allows for clear and candid communication of information to people such that they can ascertain and understand the state of affairs in a given situation and predict how their actions and actions of others will affect those affairs, and that does not unnecessarily complicate those affairs.

In the case of commercial use of health data, any data initiative or data sharing system that aims to establish and maintain the public’s trust must be willing and able to explain openly and clearly what data are being held, what proportionate privacy and security controls are imposed on them, with whom these data are being shared, and why, all reinforced and constantly updated with proper evidence.

Moreover, any data initiative or data sharing system that is centered on holding the public’s trust must be willing and able to listen to, deliberate on, and respond to the questions and comments posed by publics, and potentially incorporate their input on a meta-governance level. This of course is linked to two attributes of trustworthiness: *participation* and *accountability*. And certainly, transparency is a necessary, albeit not sufficient, condition for participation and accountability. A transparent system built on participation and accountability enables engagement in decision-making processes, allowing those whose data are collected and shared to exert some non-*de minimis* degree of influence on data sharing design and activities. To emphasize: without transparency, participation in any system is ineffective. Participation rights necessitate *a priori* transparency obligations.

Accountability is also a crucial component. Where a system of accountability is instituted, it must be accompanied by a transparency obligation imposed on the person or organization holding itself to account. Systems of accountability cannot be too complex to be understood, and cannot be designed to undermine the very purpose they set out to achieve, which is to instill confidence that those who have authority and perform *actions* will assign responsibility unto themselves, and hold themselves to monitoring, judgement, and account—with meaningful consequences—for failure to perform those actions as reasonably expected. Accountability in any data initiative or data sharing system that wants to establish and maintain the publics’ trust must be understandable and must be traceable (which is in fact an attribute of transparency). In other words, one must be able to follow the lines of responsibility throughout the course of data flow (*i.e.*, in whose hands it has been placed, and in whose hands it will be placed), and those actors involved in the flow must be able to explain and justify their conduct at all times in front of a forum that can pose questions and pass judgment, and impose consequences on those actors. What role, then, for law, human rights, and bioethics?

## 6. The Limits of Law, Human Rights, and Bioethics

Trust takes a long time to be earned and a short time to be lost. Repeated demonstration of transparency (and within it, honesty and reliability) is foundational to establishing that data initiatives, consortia, and small-scale science alike are trustworthy. Profit (even excessive profit, however it may be interpreted), commercialization, and public interest are not incompatible; opacity and public interest almost always are, however. Operating in a black box is a poor way to establish trustworthiness.

What role for law in establishing the conditions for trustworthiness? We have long relied on law and more recently, on bioethics, for good governance and protecting human rights. We should keep in mind, though, that law and bioethics, as well as science and technology, are socially constructed [22,31]. Transparency as a prerequisite for public participation in science demands a genuine recognition of the social construction of norms, be they in law or consortia ethics and science. By paying heed to the social construction of norms across time and space, and the experiences of individuals as participants in a scientific endeavor, trustworthiness can be made to stand as a viable ethico-legal normative construct that fosters equitable participation and sustainable governance of emergent new scientific practices such as consortia and collective innovation (Figure 3). As global society develops the SDGs for the next 15-year term (2016–2030), trustworthiness, by virtue of its focus on *repeated* actions of trust, resonates well with SDGs. Further, trustworthiness as a normative construct is best poised to deliver on the much discussed promises of collective innovation through just consortia.

The social construction of law and its consequential predilection to be bounded in time and space are why we are skeptical of its role in *satisfactorily* addressing the concerns raised with consortia science and ethics. Backed by the state’s enforcement structure, law can “transform messy, complex phenomena into ordered, knowable, and calculable cases, hence the momentum behind its extension” [47], but its sheer power to steer and to neutralize the stakes in any conflict [85] glosses over the inherent uncertainty and contingency of science. The law does not need to make a claim of having achieved an equitable outcome; it can be a confidence trick of social legitimacy and efficaciousness: “the rule of law depends upon public confidence and public acceptance of the system whereby [the legislature] makes the laws, the courts enforce them and the vast majority of citizens accept them until they can get them changed” [86]. As Goodrich notes [87]:

[…] law comes increasingly to dominate—to colonize—all aspects of the public sphere, it becomes the form of all political intervention in the social, and in consequence the social is depoliticized as the lifeworld comes to be structured overwhelmingly according to the agonistic and functional logic of the legal world. There is, in other words, a shift in representation of the social, a tendency to juridify all discourse and in consequence to reduce all discourse to the stable, singular […] reality of law.

In such a world of juridification, we should beware of the contrasting use of “facts” in law, which are gathered and framed internally (*i.e.*, operational and normative closure) and mobilized “in ways tailored to adjudicative needs” [88], and “facts” in the social sciences and humanities, which are gathered from observed phenomena, treated with caution and contingency, and do not follow from procedure or decree. This contrast between “epistemological modesty” [89] and “epistemological grandeur” makes us question, along with López and Lunau, whether “an over-privileging of law’s practical capacity raises the concern that more open-ended and less expedient questions will be displaced, fuelling a notion of control over technology that certainly does not correspond with our experience of handling past technologies” [47].

Further, the law tends to treat components of health research as bounded objects: data, tissue, medical devices, embryos, and so on. This fails to account for the whole, interconnected innovation system and the rich, possibly transformative, lived experiences of actors involved in health research. Law can of course facilitate (or obligate) transparency for securing the public interest, and indeed the force of law to sanction misconduct, reflected through state power, is profound. But law is often a poor enabling tool for both moral development and enforcement. It does not always facilitate transparency, and need not always, as law can all too easily be separated from questions of virtues and ethics, depending on those charged with writing or enforcing the law, or the will of the majority. Indeed, legal scholarship has shown the benefits associated with some opaque and insulated regulatory structures, including the ability to regulate unfettered by partisan politics and majoritarian preferences [90]. Law can too easily take up concern about “bad” participation, such as political posturing and inflexibility in policy positions, and “bad” accountability, such as allowing concentrated interests to monitor the actions of the decision-maker, and offer regulatory solutions of opacity that stand above the messy, political but vital participatory channels of democracy. The Janus-faced nature of law—to be both norm-creating and norm-suffocating, innovative and inflexible, and aiding and burdensome—gives us pause to take it in full embrace.

Equally, though, we wonder whether current paradigmatic (or hegemonic) conceptions of human rights and bioethics provide a more satisfactory path. Bioethics, at least in Western conceptions and as reflected in consortia ethics, remains (arguably, we admit) largely synonymous with the liberal individualism model of “respect for autonomy” [91], which is seen to represent a “synchronic and individual issue—that is, as a matter of respecting particular decisions made by an individual regarding his or her own care” at one fixed decision-making point in time [92]. Bioethics still struggles to instantiate concerns about pluralistic social, cultural, and political contexts, and how diachronic and social dimensions can arguably better interact with a respect for autonomy, which etymologically means “self-law”. The turn towards so-called “applied bioethics” has helped the philosophical and traditional strands of bioethics take up some aspects of social science research to undercover values and interests in science projects [93]. But applied bioethics is narrowly designed to ask “in frame” rather “on frame” questions, and questions remain concerning what kinds of moral claim bioethicists want to be able to make, and about normative justification and the methodological process [94]. Further, as Hedgecoe observes in his research on bioethicists’ contribution to ethical debates surrounding pharmacogenomics, bioethicists often accept unquestioningly scientists’ expectations about the development and ethical issues raised by innovations; ignore contributions from bioethicists who question these expectations; and engage in a false ethical debate, where the boundaries have been laid down and defined by academic and industry scientists [95].

In an era of global interconnectedness, where diverse cultures and peoples come together and forge dynamic links and networks, a synchronic and individualist paradigm cannot endure. The normative construct of trustworthiness can help the accustomed Western frameworks of individualist autonomy and self-law to recognize the “extended self”. In so doing, it can perhaps also facilitate the internalization of ethics norms in a “glocal” world where we need to constantly reconcile shifting global and local forces and be vigilant of where and when *not* to bend so as to protect and ensure fundamental human rights when global and local values come into conflict (Figure 3) [96].

What role for human rights? Human rights help us deliberate, but too often they remain silent in the allocation of obligations that correspond to each right. They also can fail to fully account for the unique, lived experiences of individuals, whose diverse interests and values can be lost in the course of *universalizing* rights that are balanced against each other. Human rights, especially as seen through its jurisprudence (which is where it matters most, as ultimately these rights must be adjudicated and interpreted in courts of law when they are claimed to have been violated), have become fixated on *balancing* the different interests involved in a particular case at any moment in time (often in some abstracted zone of frozen time). But fixation on balancing may promote or perpetuate injustice, especially in an age of consortia science and Big Data. Let us recall, for instance, that Big Data analytics are built on the premise that data should not be collected and used for a specific purpose or reason, but rather that they should be relentlessly collected, stored, used, and reused to find patterns, statistical correlations, and to create group profiles [97]. The purposes for their collection, their actual uses, and understanding of their value only come *after the fact*.

This should give us pause to consider whether human rights can really be balanced, and whether we should aim for balance at all, given that the harm and the interests involved are relatively vague and abstract. As Bart van der Sloot aptly notes, individuals are often unaware that “personal data are gathered and even if a person would be aware of these data collections, given the fact that data gathering and processing are so widespread and omnipresent, it will quite likely be impossible for him to keep track of every data processing which includes (or might include) his data” [98]. What real balance can be struck between the vague, protean societal interest in Big Data collection and processing, be it for national security, economic well-being, or health, and the vague, protean individual harm suffered and ambiguous individual interest attached to one’s data in a massive database? Can human rights offer guidance here? Yes, but only to an extent, and we must accept this.

And what to say of the human *duties* imposed on actors to ensure they act ethically and lawfully? As Onora O’Neill observes: “A normative view of rights claims has to take obligations seriously, since they are the counterparts to rights; it must view them as articulating the normative requirements that fall either on all or on specified obligation-bearers” [99]. This remains the open challenge to human rights that has yet to be adequately addressed by human rights’ proponents. Moreover, human rights cannot be the trump to safeguard all interests; rather, they serve as the foundation for deeper *deliberation* in our pluralistic world about what makes us connected beings, and they help us avoid extreme centrism and the risk of entrenched non-normativity that can lead to ethical and legal blind spots, including violations of human rights. To quote Michael Ignatieff fully on this point [100]:

Rights language says: all human beings belong at the table, in the essential conversation about how we should treat each other. But once this universal right to speak and be heard is granted, there is bound to be tumult. There is bound to be discord. Why? Because the European voices that once took it upon themselves to silence the babble with a peremptory ruling no longer believe in their right to do so, and those who sit with them at the table no longer grant them the right to do so. All this counts as progress, as a step toward a world imagined for millennia in different cultures and religions: a world of genuine moral equality among human beings. But if so, a world of moral equality is a world of conflict, deliberation, argument, and contention.

We need to stop thinking of human rights as trumps and begin thinking of them as a language that creates the basis for deliberation. In this argument, the ground we share may actually be quite limited: not much more than the basic intuition that what is pain and humiliation for you is bound to be pain and humiliation for me. But this is already something. In such a future, shared among equals, rights are not the universal credo of a global society, not a secular religion, but something much more limited and yet just as valuable: the shared vocabulary from which our arguments can begin and the bare human minimum from which differing ideas of human flourishing can take root.

In sum, law, bioethics, and human rights are valuable fields that should be respected and harnessed in this era of consortia science and ethics. But we must recognize their limits. Trustworthiness can work around these limitations. Trustworthiness fosters virtuous behavior in the health context and views opacity as a disadvantage and transparency as an unalloyed good. It reaches beyond but also operates at the interface of law, human rights, and bioethics. By considering trustworthiness as a central pillar at the intersection of law, human rights, and bioethics, we enable others to trust us, which in turns allows different actors (both nonprofit and for-profit) to access and responsibly use health data for public benefit and to operate more justly.

## 7. Conclusions

In this article, we have suggested that today’s Big Science projects, be they the whole-genome sequencing of a population, population-based biobanks, high-energy experimental physics or space exploration, are characterized by the intertwining and embedding of ethicists and scientists in large-scale global consortia. In the course of this intertwining is the centripetal force of extreme centrism. Common sense holds that large units like consortia, comprised of diverse value structures and geographies, will adopt a more centrist position and posture. This comes at a cost, though, and in fact can be a radical posture; it is not always traditionally extreme (read: hard left or right) ideas that are the most radical, but rather those that tempt to invariably stay in the middle normatively to keep diverse norms, interests, and methods together for a functionalist (read: science enabler) ethos.

The intensively populist rhetoric in consortia can mask the emergence of extreme centrism. The time is ripe for theorization and empirical study of extreme centrism in the context of Big Science, Big Data, and Big Ethics. For every first order action there is a second order consequence, and what may appear as mundane can in fact have radical consequences. Little to date has been said about these effects on “little science” and “indie” ethics, compared to the benefits of Big Data and ever larger science and intertwined ethics. As consortia science and ethics encourages a pull towards the extreme center, there may be a risk for not taking action when one should. Our article attempts to inject dialectical symmetry into the discussion of the big domains in science, ethics, and data. We recognize that our discussion is conceptual and more question-raising, and we therefore encourage that the impact of big funding and consortia science on bioethics as a discipline and profession, and its collateral consequences on human rights, be studied empirically—and herein lies the role of the truly independent bioethics (or social science) scholars who can act as second-order narrators [76,77].

In order to effectively address the legitimate concerns about commercial use of health data and the rapid rise of consortia science and ethics, we should consider that the common goal in both is neither to “safeguard public confidence” in “appropriate” uses of health data, nor to prevent the sharing of ideas and capital and the co-production of knowledge. Rather, the common goal is to foster virtue, to establish conditions of trustworthiness in all systems governing use of health data, and in all systems working on large-scale scientific endeavors that inevitably raise ethical questions. By instituting effective transparency through policy and conduct, we show that we are trustworthy. Even in an era symbolized by Big Data, Big Science, and Big Ethics, local relationships and local values still retain power [96]. Relationships defined by respect, trust, and mutual obligation can make the difference between success and failure, and between human health innovations and human health setbacks.

While discussions about global science governance remain fluid, it would do us well to recognize trustworthiness as a central pillar of a robust governance system, and a virtue to be instilled in every person. It would allow others to come to trust us, which in turns would allow diverse actors to use health data for the public benefit and to co-produce knowledge for the betterment of humanity, however so interpreted and applied in the agora of our “glocal” communities [96].
